# Enhanced Heterosexual Transmission Hypothesis for the Origin of Pandemic HIV-1

**DOI:** 10.3390/v4101950

**Published:** 2012-10-03

**Authors:** João Dinis de Sousa, Carolina Alvarez, Anne-Mieke Vandamme, Viktor Müller

**Affiliations:** 1 Laboratory for Clinical and Epidemiological Virology, Rega Institute for Medical Research, Katholieke Universiteit Leuven, Leuven B-3000, Belgium; Email: joao.sousa@rega.kuleuven.be (J.D.S.); annemie.vandamme@uz.kuleuven.be (A.-M.V.); 2 Instituto de Medicina Tropical Alexander von Humboldt, Universidad Peruana Cayetano Heredia, Lima 31, Peru; Email: carolina.alvarez@upch.pe (C.A.); 3 Centro de Malária e Outras Doenças Tropicais, Instituto de Higiene e Medicina Tropical, Universidade Nova de Lisboa, Lisboa 1349-008, Portugal; 4 Research Group of Theoretical Biology and Evolutionary Ecology, Eötvös Loránd University and the Hungarian Academy of Sciences, Budapest 1117, Hungary; Email: mueller.viktor@gmail.com

**Keywords:** HIV, SIV, Origin of HIV, zoonosis, Central Africa, genital ulcer disease, unsterile injections, bushmeat

## Abstract

HIV-1 M originated from SIVcpz endemic in chimpanzees from southeast Cameroon or neighboring areas, and it started to spread in the early 20th century. Here we examine the factors that may have contributed to simian-to-human transmission, local transmission between humans, and export to a city. The region had intense ape hunting, social disruption, commercial sex work, STDs, and traffic to/from Kinshasa in the period 1899–1923. Injection treatments increased sharply around 1930; however, their frequency among local patients was far lower than among modern groups experiencing parenteral HIV-1 outbreaks. Recent molecular datings of HIV-1 M fit better the period of maximal resource exploitation and trade links than the period of high injection intensity. We conclude that although local parenteral outbreaks might have occurred, these are unlikely to have caused massive transmission. World War I led to additional, and hitherto unrecognized, risks of HIV-1 emergence. We propose an Enhanced Heterosexual Transmission Hypothesis for the origin of HIV-1 M, featuring at the time and place of its origin a coincidence of favorable co-factors (ape hunting, social disruption, STDs, and mobility) for both cross-species transmission and heterosexual spread. Our hypothesis does not exclude a role for parenteral transmission in the initial viral adaptation.

## 1. Introduction

Simian Immunodeficiency Viruses (SIV) have jumped to humans at least thirteen times, leading to four known groups of Human Immunodeficiency Virus (HIV) type 1 (HIV-1) and nine groups of HIV type 2 (HIV-2) [1,2,3,4,5,6,7,8,9,10,11,12]. Both the pandemic strain (HIV-1 group M) and a rare variant only found in Cameroon (HIV-1 group N) originated from SIVcpz infecting wild chimpanzees of West Central Africa (*Pan troglodytes troglodytes*) [1,2,3,4,5]. The rare HIV-1 group P, also found in Cameroon seems to have originated from SIVgor infecting western lowland gorillas (*Gorilla gorilla gorilla*) in the same region [10,11]. HIV-1 group O, which infects thousands of people mainly in Cameroon and neighboring countries, has SIVgor as its closest known relative, but an origin from SIVcpz cannot be formally excluded [1,13,14]. All known nine HIV-2 groups are derived from SIVsmm endemic in sooty mangabey monkeys from the West African forests. Only HIV-2 groups A and B generated substantial human epidemics [4,5,6,7,8,9].

It is widely accepted that the most likely route of simian-to-human transmission of SIV was through bushmeat hunting and handling [2,3,4,9,15,16,17,18]. One study reported a prevalence of seroreactivity to SIV of 17% in Central African villagers highly exposed to simian bushmeat, significantly higher than in unexposed individuals [19]. Other simian retroviruses have found their way into humans through similar bushmeat practices, such as simian foamy virus [20] and simian T-cell lymphotropic virus (STLV) [21,22]. Only a few of such zoonotic infections have turned into the human epidemics of HIV and HTLV.

Molecular clock analyses were used to estimate the timings of the most recent common ancestor (tMRCA) of epidemic human retroviruses. Estimates of tMRCA for the different HTLV-1 subtypes span between less than 3,000 and about 50,000 years ago [23,24]. HTLV-2 crossed to humans between 60,000 and 400,000 years ago [22,23,24]. In contrast, the tMRCAs of all four main epidemic HIV groups (HIV-1 groups M and O and HIV-2 groups A and B) appear to be all very recent and nearly simultaneous in the early 20th century [6,25,26,27,28,29,30,31]. Similar research showed that the tMRCA of HIV-1 group M and its closest SIVcpz relative found to date, predates the spread of HIV-1 group M by only a few decades, suggesting that the cross-species transmission of SIVcpz giving rise to HIV-1 group M must have been in late 19th–early 20th century. This fact suggests that phenomena new in the 20th century might have constituted the driving factors of HIV emergence. Among the potential factors, it has been noted that bushmeat intensification, forced labour, and smallpox vaccinations performed without proper sterilization were common in colonial Afrique Équatoriale Française (AEF; a federation of French colonies encompassing French Congo, Gabon, Oubangui-Chari, and Chad), these factors potentially increased human exposure to SIV and risks of human-to-human transmission and viral adaptation [5].

Several authors proposed a major role of unsterile injections in HIV origins. One hypothesis proposes that unsterile injections serially transmitting SIV between humans in a chain of 3–4 acutely infected people, improved its adaptation to the new host (as happens in experimental serial passage of SIV in macaques and monkeys [4]). In this hypothesis such serial transmission chains are rare events, but once one happens the resulting adapted virus has long term epidemic potential, even if there is no further transmission immediately after the adaptive event [4,32,33,34]. The critical injections that might have fostered the origin of the several HIV groups were hypothesized to be either those of the antibiotic era (after ~1950) [4,32] or those belonging to the campaigns against human African trypanosomiases (HAT) (which peaked in 1925–1945) [34]. 

Another parenteral hypothesis proposes that unsafe injections used in the treatment of tropical and infectious diseases (e.g., HAT, yaws, syphilis) might have massively transmitted SIV/HIV in rural areas, generating large hubs of infected people, with sexual transmission gaining importance only later on [17,18]. It was proposed that HAT treatments performed in southwestern Oubangui-Chari (now the Central African Republic (CAR)) exponentially transmitted HIV-1 group M among the Mbimou tribe in the 1930s–1940s, causing a large proportion of them to die of AIDS [35]. 

Social changes, and/or increased human mobility [3], and the onset of urbanization in colonial Africa [27] have also been invoked as explanations for HIV emergence. We proposed that a high frequency of genital ulcer diseases (GUD), such as syphilis, chancroid, and lymphogranuloma venereum (LGV), a result of those social changes, and possibly also a relatively low frequency of male circumcision, in nascent colonial cities may have been the triggers of initial HIV transmission and adaptation to humans [16]. GUD induced genital ulcers can dramatically increase HIV transmission [16,36,37], and our work suggests that the high prevalence of these diseases might have provided crucial “help” for the initial spread of the virus.

In this article, we will focus on the origin of HIV-1 group M (HIV-1 M). Studies of sera collected in the period 1959–1992 (reviewed in [38]), the history of the early African AIDS cases (reviewed in [39,40]), the high genetic diversity exhibited by HIV-1 M in the Democratic Republic of Congo (DRC) compared to other African countries, particularly in Kinshasa [27,41,42], and a recent phylogeographic analysis using HIV-1 M strains from many different DRC locations and from Brazzaville [43] suggest that, by 1950, Kinshasa was the main human epicenter of the epidemic [2,15,16,18,27,43]. Thus, this city may have been the place where the virus underwent its main initial spread and diversification, whether or not early foci first occurred elsewhere. 

HIV-1 M clusters most closely with SIVcpz currently found in southeast Cameroon [2], which suggests that the cross-species transmission happened in this part of Africa [2,15]. This motivated us to investigate which factors present in these areas in the early 20th century might have been able to potentially promote SIV zoonotic transmission, sexual or parenteral transmission of the virus between humans, and migration of the virus to major centers. We retrieved colonial reports and archival data about Cameroon and AEF, and additional colonial medical, ethnographic and other literature to compare the different theories of HIV origins with available information from such archives.

## 2. Study of Risk Factors

### 2.1. The Patchy Prevalence of SIVcpz and SIVgor

All known HIVs that originated in Central Africa are derived from either SIVcpz or SIVgor. The chances for an HIV-1 epidemic to originate in a particular region within the ranges of the relevant chimpanzee and gorilla subspecies would depend on local SIVcpz/SIVgor prevalence in wild apes, levels of ape bushmeat hunting, and the chance that an infected human carrier would bring the virus to a city hub at or near these areas, where transmission factors were favorable to its initial adaptation and spread. Most regions watered by the navigable branches of the Congo-Oubangui-Sangha fluvial system (which therefore had more contacts with the above cities during the early colonial period) are inhabited by either central chimpanzees (*P.t.troglodytes*), eastern chimpanzees (*P.t.schweinfurthii*), or western lowland gorillas (*G.g.gorilla*), the reservoirs of SIVcpz and SIVgor. SIVcpz seroprevalence is very patchy, with most communities having zero or low seroprevalence, and a minority with a prevalence around 30%–60% [2,44,45]. SIVgor seroprevalence is only 1.6% on average and is zero at most sites [13]. Ape bushmeat hunting was and is widespread in Central Africa, but its intensity varies across regions [16,46,47,48].

### 2.2. The Region under Study

**Figure 1:**
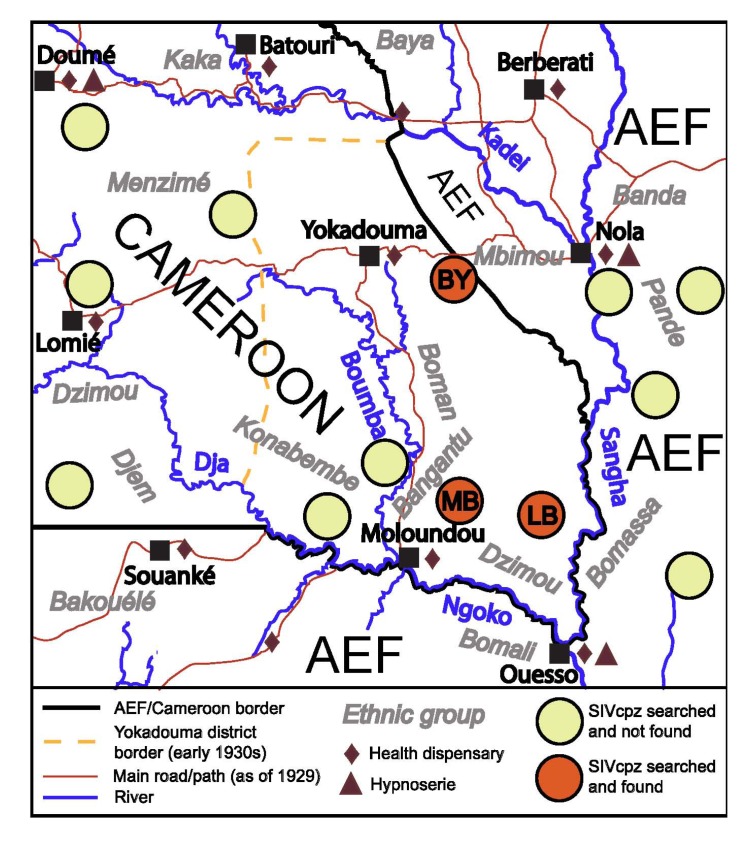
The region of southeast Cameroon and neighboring Afrique Équatoriale Française (AEF) regions circa 1930. Sources: (1) on the SIV surveys: [2,13,15]; (2) on other elements: [50,51,52,53].

HIV-1 M is most closely related to SIVcpz retrieved from the southeast corner of Cameroon, a region flanked by the Sangha, Ngoko, and Boumba rivers, from the sites MB and LB (Figure 1) [2]. SIVcpz forms phylogenetic clusters associated with regions separated by rivers and other geographical barriers, which pose obstacles to chimpanzee migration [2]. This, and the short branches connecting HIV-1 M to SIVcpz collected from this area, justify the assumption that this group likely originated in the area enclosed by the above mentioned rivers [2,15]. Here we will build on this assumption, while recognizing that it would need to be revised, should future evidence indicate that chimpanzee (or its virus) migration was more extensive over the century since the origin of HIV-1 M. In this region, current SIVcpz prevalence in chimpanzees is 20%–35%, higher than in chimpanzees from Cameroon and Gabon as a whole [2,44,49], while hundreds of local gorilla fecal samples did not yield any evidence of SIVgor infection [13].

### 2.3. Evolution of Ape Hunting Practices over Time

Most Central African peoples that share their habitat with apes tend to hunt them, although there are important exceptions [16,46]. For the studied area, we gathered numerous references documenting the hunting of apes throughout the colonial period and beyond, both within the Boumba-Ngoko-Sangha enclosure [46,54,55,56,57,58,59], and in adjacent regions west of the Boumba [46,57,60], south of the Ngoko [46,59,61], and east of the Sangha [61,62,63,64,65].

According to some sources, the Aka and Baka pygmies that inhabit these regions seldom eat ape meat, although they hunt them to supply meat to clients [59,65,66]. However, at least in some of the areas, pygmies eat ape meat frequently [63,64]. The non-pygmies of these areas generally eat apes, although this is restricted by certain beliefs: often an animal species is thought to represent the clans’ ancestors, or to have helped them in the past, this preventing their hunting. For example, gorillas and chimpanzees are not killed by a branch of the Bangantu ethnic group, because they are believed to have helped their ancestors [67]; some Djem clans regard the gorilla as their totem, and do not hunt these animals [60]. It should be kept in mind that clans are often more important than ethnic groups as determinants of hunting restrictions [68]. 

In many of the ethnic groups of the region, women were traditionally forbidden to or strongly discouraged from eating chimpanzee and gorilla meat [46,47,48,54,56]. Gorilla meat is generally preferred to chimpanzee meat [46,48,63,64].

In 1907, a French observer noted that the Mbimou people, who live between Yokadouma (Cameroon) and Nola (AEF), “loved” to hunt chimpanzees and gorillas. Colonial concessionary companies corroborated this [55,56]. Chimpanzee hunting for bushmeat was probably practiced in the area around Moloundou in 1910–1912, because a German observer remarked that chimpanzee meat was forbidden to women [54]. In the 1930s, the hunter Fred Merfield noted that the Menzime hunted gorillas more frequently than the peoples of south-central and southwest Cameroon [47].

French and German concessionary companies sold thousands of firearms in the region in exchange for rubber and ivory in the period 1895–1910, and observers noted that most local peoples (Bangantu, Konabembe, Menzime, Mbimou) owned firearms [46,47,54,55,56,69]. More than 10,000 had been sold by the French Compagnie Ngoko-Sangha (CNS) in the period 1900–1907 [69]. These were mostly old‑fashioned muzzle-loaded guns (although modern rifles were also sold), with which the hunting of apes is difficult and unsafe for a lone hunter. However, it was observed that muzzle-loaded guns were used by hunters, jointly with spears and other traditional weapons, to kill gorillas in collective round‑ups [46,47]. From these descriptions, it is unclear whether muzzle-loaded guns contributed greatly to the efficiency of ape hunting; however, villagers with any type of guns may have been more willing to hunt, which probably led to more ape hunting.

Ivory collection was intense in this region in the first decade of the 20th century: already by 1912–1914 there were worries about the extinction of elephants in the region [56]. Rubber collection was forcibly imposed on villagers by colonial companies in exchange for low pay. They had to move deeply into the forests, staying separated from their families for weeks, and this increased reliance on bushmeat [56,57,70]. The Menzime specialized in supplying bushmeat to rubber collectors and hunted apes very frequently [47]. In the early to mid-1920s, the lower post-war demand and increased competition from South-American and Asian rubber caused the local rubber prices to collapse [50]. This caused the foreclosure of most enterprises of the Mouloundou area in 1922–1923 [47,50]. However, a new economic development wave centered on coffee and cacao plantations started at that time, and the plantation workers were supplied with bushmeat by specialized hunters [56]. Animal skins were procured by concessionary companies and rubber collection continued, albeit reduced. In addition, men working in road construction (more important since the late 1920s) may have been supplied with bushmeat too, since this was common in Central African enterprises [71,72]. These activities probably maintained a high level of human exposures to SIVcpz and SIVgor even after the end of the “rubber and ivory” period. 

From 1916 onwards, the AEF administration introduced measures to protect wildlife, including elephants and gorillas (but not chimpanzees) [56]. New hunting laws and permits were enacted in 1929–1930, in both AEF [56,73] and Cameroon (report of the French administration of Cameroon to the League of Nations, 1930 [50]). Chimpanzee hunting became restricted to holders of scientific permits. Gorilla hunting became restricted to holders of either scientific or “*sportif*” (touristic) permits (who were almost always European), and holders of special large game permits (which could be privileged Africans). 

The enforcement of these laws was difficult in interior areas, where colonial administrative personnel visited less frequently. Accordingly, we read numerous descriptions of ape hunting in which the law was violated: Merfield observed collective round-ups of gorillas practiced by the Yaunde and the Menzime in Cameroon, in the late 1920s and 1930s [47]; Denis observed such practice among the Mbeti (French Congo) in the 1940s [46]. In the Belgian Congo, where similar laws protecting apes existed [74,75], live infant chimpanzees were being traded in the 1950s [39,40]; this implies that their parents had been killed [39,76], at a time when colonial administrative control was at its peak.

With decolonization, in 1960, hunting law enforcement degraded even further, modern firearms became more available, and bushmeat became increasingly commercialized, accelerating the collapse of the chimpanzee and gorilla populations (worldwide, chimpanzees were between one and two million in 1960, and had declined to 170,000–300,000 in 2003 [77,78,79]). We can tentatively suggest that ape hunting was less intense in the period 1930–1960, when the relevant protective laws were being partly enforced, than either before or after that.

### 2.4. Nosocomial Risks

#### 2.4.1. General Trends

Parenteral risks such as posed by unsterile injections and vaccinations could have favored initial HIV spread in both rural areas and cities. In this article we attempt to quantify these risks in the rural area under study, while recognizing that quantifying them in the colonial cities more probably associated with early HIV spread (e.g., Kinshasa) is a worthwhile project. 

**Figure 2:**
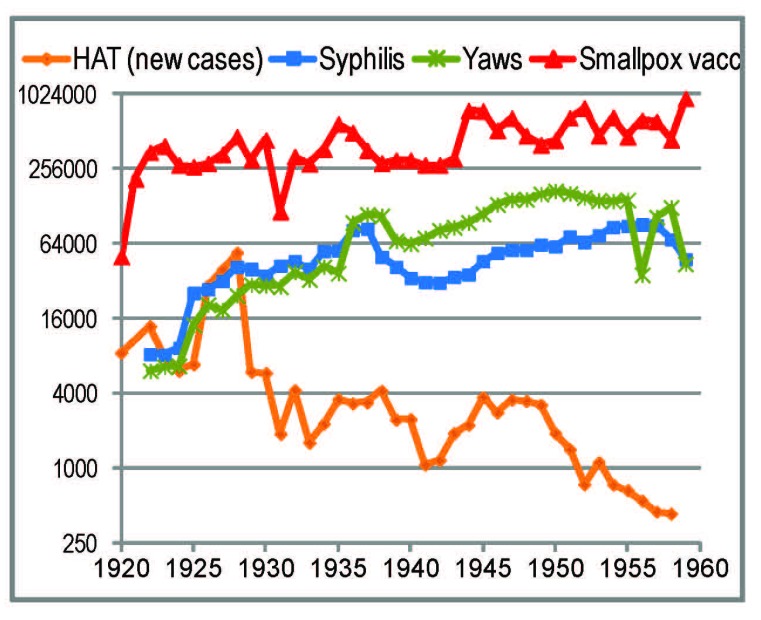
Reported annual numbers of treatments against human African trypanosomiasis (HAT) (only newly diagnosed cases), syphilis and yaws, and of smallpox vaccinations in French-administrated Cameroon, in the period 1920–1960. The references are listed in Methods.

Several reviews indicate that the broadest (in the sense of number of people covered) healthcare interventions involving injections/inoculations in rural Central Africa up to Word War II were smallpox vaccinations and treatments against HAT and treponemal diseases (yaws and syphilis) [17,18,34,72]. Figure 2 displays the annual numbers of these interventions in Cameroon.

#### 2.4.2. Smallpox Vaccinations

Colonial medical authors had expressed concern about the parenteral risks of smallpox inoculations from very early times. One, writing in 1906, reported that catheters were carefully boiled between inoculations of *Tirailleurs* (the elite African troops) [80]. In vaccinations of the general African population the standards of safety were probably lower, although several sources of the early 1920s do mention sterilization procedures [81,82], such as plunging catheters in a saucer with burning alcohol [82]. Such “flaming” procedures do effectively kill retroviruses like SIV or HIV; however we do not know how often vaccinations were performed with inadequate or no sterilization procedures.

Jennerian inoculations were practiced in Cameroon and AEF throughout the colonial period. They were often ineffective, and this prompted medical authorities to revaccinate people often. In the period 1922–1940, the annual inoculation rate in the population of French-administrated Cameroon was in the range 12–22% [50], so each person was being inoculated once every 5–8 years. In AEF, the frequency was slightly lower [51,72]. This frequency of parenteral exposures was much lower than in injection treatments (the latter will be reviewed below).

In the period 1890–1910 there were shortages of viable vaccine in interior regions because the heat during the long journey to the interior often caused them to lose effectiveness. Pressed by smallpox epidemics, doctors performed arm-arm inoculations: they collected lymph from the pustule of a recently vaccinated person to vaccinate the next. As was well recognized at the time [81], this procedure causes a much higher parenteral risk because more lymph is transferred than in normal unsterile catheter reuses. Some of these interventions were done by the French in “Haute Sangha”, which normally designates the region to the north of (and including) Nola [80,83,84]. Lymph was collected from relatively few individuals, each donating to many people, and lymph was gathered from their pustules 4–5 days after inoculation [84,85,86]. This practice was, however, less common than the normal method of Jennerian inoculation. 

#### 2.4.3. Yaws and Syphilis Treatments

In Cameroon, anti-treponemal treatments rose sharply in 1925. They multiplied 10-fold between the early 1920s and the 1950s [50,87,88,89,90,91,92,93,94,95,96,97] (Figure 2), *i.e.*, with a peak later than the origin of the more widespread HIV-1 groups (M and O). In forested areas (as in the region where HIV-1 M came from) yaws was more common than syphilis. In the 1920s and 1930s, 90%–95% of people treated for yaws were children [94,98]—less likely to be SIV- or HIV-infected.

The most important drug used against these diseases was the arsenical compound *neoarsenobenzol* (called *neosalvarsan* or *neo*), available since 1912, soon after its predecessor *arsenobenzol*. It was administered intravenously. Other arsenical compounds, and bismuth and mercury compounds were also used. Bismuth was administered intramuscularly or subcutaneously [99,100,101] and, being better tolerated by Africans than mercury, became increasingly preferred [102]. Orally administered *stovarsol* was also heavily used. To attempt complete cures of syphilis, prolonged treatment regimens involving many injections (combining an arsenical with other compounds) were recommended [103]. Accordingly, the best anti-venereal disease hospitals, clinics, and dispensaries in urban centers often administered many injections per patient. For example, in the Institut Policlinique de Dakar, in 1933 and 1934, syphilis patients received an average of 17 and 25 injections per year, respectively; in the Vernes Dispensaries of Madagascar, in 1934, the average was 17 [94]; in the Croix Rouge du Congo of Leopoldville-East, the averages of injections per syphilis patient per year, in the period 1933–1940, were in the range 15–24 [101]. 

However, in less favored dispensaries and in rural areas, much fewer injections were given [92,94]. For example, in non-specialized Madagascar dispensaries, in 1934, the average was 3.7 injections per syphilis patient per year, of which 1.25 were of arsenicals [94]. We refer these Madagascar data because only for this colony the French articles we reviewed provide the figures of injections per patient for "normal" (non-specialized in venereal disease) dispensaries. This is relevant, because the dispensaries existing in the region under study were also non-specialized. Several authors writing about syphilis and yaws treatments in medical *tournées* by rural areas of AEF and Cameroon in the 1920s and 1930s, state that the average patient received just one or two *neosalvarsan* injections [98,92,104]. This is corroborated by the administrative reports of Cameroon sent to the League of Nations: in the period 1922–1931 the average number of such injections per treponematosis patient per year was in the range 1.0–1.9 (or 1.4–2.3 counting mercurial and *sulfarsenol* injections) (Table 1) [50,88,105].

**Table 1:** Injection treatments against treponematoses in Cameroon, 1922–1931. Sources: [50,88,105].

Year	# of treponematoses cases		# of injections			# of injections/patient/year	
	Syphilis	Yaws	Neosalvarsan	Mercury salts	Sulfarsenol	Neosalvarsan	Others
1922	8,285	6,129	27,800	NA	NA	1.93	NA
1923	8,237	6,552	20,050	NA	NA	1.36	NA
1927	32,303	18,930	59,200	15,274	22,310	1.16	0.73
1928	42,015	24,718	76,270	18,158	59,450	1.14	1.16
1929	40,341	30,031	75,567	20,128	4,731	1.07	0.35
1930	35,693	29,701	77,728	23,085	15,591	1.19	0.59
1931	42,730	29,086	106,669	27,800	32,680	1.49	0.84

NA: Not available.

#### 2.4.4. The HAT Campaigns

Two drugs constituted the main arsenal against HAT (sleeping sickness) in Central Africa, until the Second World War: *atoxyl*, administered intramuscularly, which had little therapeutic value, and *tryparsamide*, administered intravenously, which permitted cures in advanced disease. Other intravenous drugs were used in the 1930s, but their combined number of injections was considerably lower than that of *tryparsamide*. In AEF, Belgian Congo, and German Kamerun (we will use this spelling to refer to Cameroon under German colonization, 1884–1916), *atoxyl *started to be used around 1907–1909 [72,106,107]. Colonial doctors believed that *atoxyl *could clear the peripheral blood from trypanosomes and thus, injecting rural people on a large scale would prevent the spread of the epidemic. They performed numerous “*tournées d'atoxylization*” in many rural areas of Central Africa in the two decades following 1907, administering usually one or two injections to each person infected or suspected to be infected [72,108,109,110,111,112]. Several injection campaigns were performed by the French in Haute Sangha (including Nola) in the period 1907–1913 [83,109,112]. Others were performed by the Germans in the Nola region (which was annexed to Kamerun in the period 1911–1914 following a Franco-German agreement) and in neighboring southeast Kamerun, where the disease was observed on the banks of the Ngoko [72,113]. These *tournées* were very irregular: some interior regions were not visited at all for many years; and, particularly in the 1920s, some regions were visited often, causing some people to receive six and sometimes more injections per year [72,114].

*Tryparsamide *started to be used in the mid-1920s, and was administered in series of weekly intravenous injections (with varying regimens). HAT campaigns peaked earlier than syphilis and yaws treatments, *i.e.*, nearer to the calculated tMRCAs of HIV-1 groups M and O [26,27,28,29,30]. In Cameroon, they had a sharp peak in 1926–1928 (Figure 2), when Eugene Jamot started more organized campaigns based on mobile teams that visited most of the population [50,115,116,117,118]. A few years later, similar teams were put in place in AEF [72,119]. Review articles about these organized campaigns of French Cameroon and AEF show that each patient received 15–24 injections, spread over a period of treatment of two or more years. More than half of the injections were of the cheaper and non-intravenous *atoxyl *[115,116,120]. 

This suggests that the organized HAT campaigns, during their peak period in Cameroon and AEF (broadly 1926–1940), involved considerably higher parenteral risks than syphilis and yaws treatments in the same regions. Probably, the early “*atoxylization*” campaigns (1907–1926) posed lower parenteral risks than the more organized ones (which started in 1926): they were irregularly done, each patient received relatively few injections, and these were intramuscular or subcutaneous.

Colonial health reports mention the number of HAT patients surveyed (newly found ill or old patients revisited). All new patients, and some of the old ones were treated. In 1930, the surveyed HAT patients in AEF received on average only 3.05 injections each (the average of those *effectively treated* was higher; we cannot calculate it because the proportion of surveyed old cases treated was seldom quantified in the reports). This average increased throughout the 1930s (Table 2). 

**Table 2:** Injections against human African trypanosomiasis (HAT) in Afrique Équatoriale Française (AEF). All “new” cases were usually treated with around 12 injections; however, in 1930, this was probably less, given the total number of injections reported. Not all surveyed “old” cases were treated. Sources: [90,94,95,97].

Year	# of surveyed HAT cases		# of injections performed	# of injections per patient per year		
	New cases	Old cases		All surveyed cases	New cases (all treated)	Old cases
1930	12,649	34,611	144,168	3.05	6-8?	1-2?
1934	13,368	42,508	254,000	4.55	~12	2.20
1935	12,557	54,709	262,424	3.90	~12	2.75
1937	16,597	60,196	597,409	7.78	~12	6.62

#### 2.4.5. The Early 1930s: Intensification of Injection Treatments

The organized HAT campaigns in Cameroon started in 1926, and the number of newly identified patients peaked in the period 1926–1928 (Figure 2). However, this effort did not arrive to the southeast district of Yokadouma (which encompassed the Cameroonian banks of the Ngoko and Sangha river; Figure 1) until 1932, when this area was first visited by the mobile teams [50,92]. The teams, which visited almost all inhabitants, found 14% of them infected (44% in the southern areas near the Ngoko) [50]. Each infected person may have been treated with more than 15 injections in the next few years (see above). In addition, the report of Cameroon for that year [50] states that the district never had continuous medical action before. Thus, the year 1932 brought unprecedented levels of parenteral risks to southeast Cameroon.

Anti-HAT campaigns in the neighboring AEF regions of Nola and Ouesso peaked after 1929–1930, although there was some activity before [72]. In Nola, there were only 380 new HAT cases treated in 1930, and the number rose sharply after that (Table 3). The Mbimou people, who live between Yokadouma and Nola were the main focus but were only systematically treated in the 1930s (Table 3 and related references). However, according to a reviewer, they were being insufficiently treated at least until 1935 [121]. Our own research of the relevant AEF reports [51] reveals that each surveyed (new or old revisited) HAT patient from Nola (mostly Mbimou) received an average of 4.58 injections in 1933 and 4.59 the following year.

**Table 3:** Systematic treatments against human African trypanosomiasis (HAT) in southeast Cameroon and neighboring districts of Afrique Équatoriale Française (AEF), up to 1936. Dashes indicate no systematic activity, according to the reports. Some of the old cases were also treated. Sources: [50,51,87,88,89,90,91,92,93,94,95,96,97].

Year	Yokadouma sector ^a^ (Cameroon)		Nola sector (AEF) ^b^		Ouesso sector (AEF) ^b^	
	New cases	Surveyed old cases	New cases	Surveyed old cases	New cases	Surveyed old cases
1927	–	–	–	–	–	–
1928	–	–	228	222	108	?
1929	–	–	77	176	212	242
1930	–	–	380	?	154	356
1931	–	–	594	2,371	116	457
1932	2,928	–	590	1,812	130	957
1933	?	?	885	3,409	177	1,632
1934	43	2,928	1,463	2,516	184	818
1935	?	?	1,816	7,342	b	b
1936	74	1,036	3,464	3,721	b	b

^a^ Yokadouma was included in the Boumba-Ngoko sector. ^b^ Ouesso was included in the Ngoko‑Sangha sector up to 1934, and in the Sangha sector (jointly with Nola) after that.

Therefore, the early 1930s brought unprecedented levels of parenteral activity to southeast Cameroon and neighboring AEF areas: not only were anti-HAT campaigns systematically performed by then, but the same mobile teams also boosted anti-syphilis and anti-yaws treatment.

**Figure 3:**
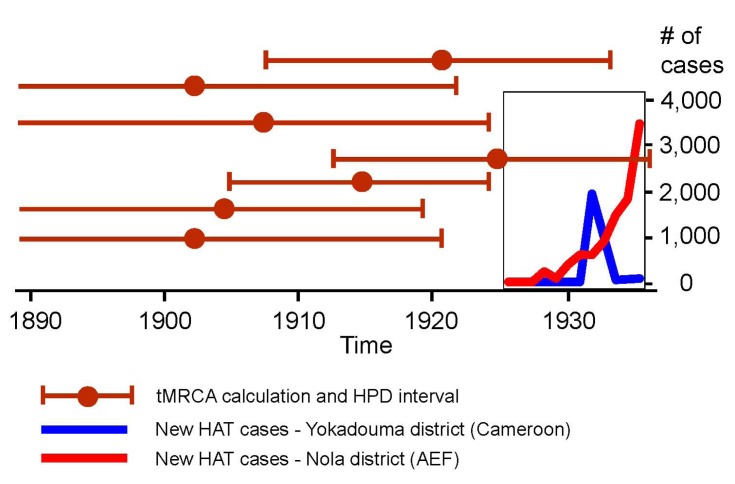
The recent tMRCA calculations of HIV-1 M (with high probability (HPD) distribution intervals), and the timings of systematic treatments against human African trypanosomiasis (HAT) in the districts of Yokadouma (Cameroon) and Nola (Afrique Équatoriale Française). Sources: (1) on tMRCA calculations: [27,30]; (2) on HAT treatments: [50,51,87,88,89,90,91,92,93,94,95,96,97].

Most calculations of HIV-1 M’s tMRCA published recently produced results in which the upper interval of the highest probability distribution falls before 1930 (with a few exceptions) [27,30]. This is illustrated in Figure 3, and suggests that HIV-1 M probably originated at a time when parenteral risks in the studied region were considerably smaller than in the 1930s.

#### 2.4.6. Health Facilities

During the German period, there was an important medical facility in Moloundou [122]. In the 1920s and early 1930s, Moloundou and Yokadouma had only dispensaries run by nurses [72]. In the neighboring AEF areas, only Ouesso and Nola had a doctor each until 1934, whereafter a third medical post, in Berberati, is mentioned in the reports [51]. In the 1920s and early 1930s, there were a few nurse-operated dispensaries in Souanké and Sembé (west of Ouesso) and in Berberati and Carnot (north of Nola). In 1931, a new one was set up in Gamboula, at the AEF-Cameroon border [51]. During these decades, segregation camps for HAT patients (*hypnoseries*) existed in Ouesso and Nola (the latter since 1909). Leprosariums were set up in Nola and Berberati in the early 1930s. In addition to these facilities, missions provided some healthcare [50,51,72,121]. In these facilities, the number of injections per patient in HAT, syphilis, and yaws treatments was probably higher than in rural villages.

#### 2.4.7. Comparison with Modern HIV Parenteral Outbreaks

In an HIV-1 outbreak in Romanian orphanages, children received around 147 injections per year (according to a detailed study based on one of the orphanages) [123]. Estimates for injection intensity in nosocomial outbreaks in Elista, Russia, and in Lybia give an annualized rate of 40–200 and 52, respectively; in both of the latter outbreaks, HIV-1 transmission was probably boosted by the use of venous catheters and lines in many patients; no reliable estimates exist for the other outbreaks [124]. 

Studies of intravenous drug users (IDU) estimated that they inject themselves around ~700 (300–1,800) times a year [125,126,127,128,129,130,131]. In Western IDUs, explosive HIV-1 spread happened more commonly in the 1980s (with prevalence attaining 15%–70%), when they shared needles more frequently and needle exchange programs, methadone, and other harm reduction interventions were still incipient or non-existent [125,132,133,134,135,136]. For IDUs observed at the start, or in early stages, of these interventions the average frequency of needle sharing (counting all individuals including non-sharers) was normally in the range 60–245 needle sharing events per year (and much higher counting sharers only) [125,131,132,133,137].

While in some IDU communities the sharing frequency remained similarly high despite interventions [126,127], in others the interventions were very successful in reducing the needle sharing frequency to 10–30 per year (and sometimes to <10) [129,130,135,138]. In the latter cases HIV-1 prevalence either is very low, or is persistently stable or declining. For example: (1) in Australia, interventions were very effective, annual needle sharing rate is <10, and HIV-1 prevalence is stable at ~1% [129]; (2) in Edinburgh’s IDUs, after explosive HIV-1 spread in the early 1980s, effective interventions reduced needle sharing rate to <20 per year, and HIV-1 prevalence remained stable around 20% during many years [138]; (3) a similar pattern was observed in several Dutch cities (estimated annual needle sharing rates of ~23 in Rotterdam and ~14 in Arnhem) [135], and in Montréal (estimated annual needle sharing rate of ~27 [130]; (4) in Vancouver, HIV-1 prevalence was stable at 3–4% in the period 1984–1994, and annual needle sharing rate was estimated at ~30 [139]; (5) the IDUs from Bristol, UK, were estimated to share needles 48 times per year and had only 1% HIV-1 prevalence, while those from Teesside, UK, shared needles 14 times per year and had zero HIV-1 prevalence [140]. Such data seem to suggest that the above sharing frequencies do not enable HIV-1 rapid spread in IDUs.

**Figure 4:**
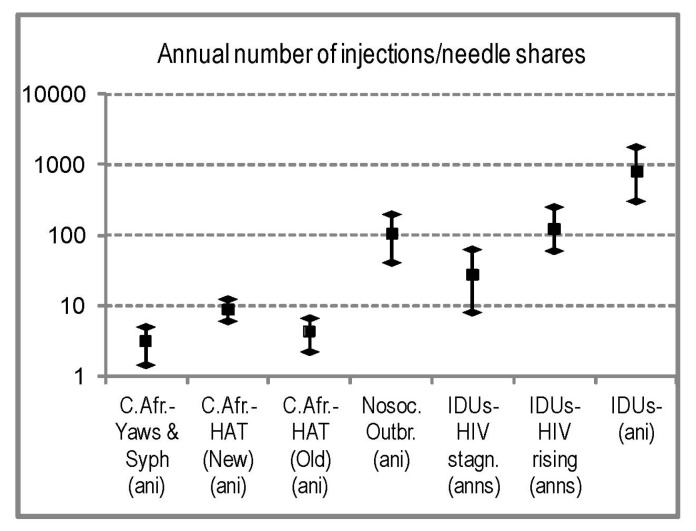
Comparison of injection intensities in the Central African campaigns (first three categories) and relevant modern populations (remaining categories). (ani): annual number of injections; (anns): annual number of needle shares. The seven categories, from left to right, are as follows: (**i**) to (**iii**) Central African data, based on our review (Table 1 and Table 2); (**iv**) available estimates for nosocomial outbreaks (sources: [123,124]); (**v**) estimates of needle sharing for IDU groups in which HIV-1 prevalence was either <5% or stagnant since many years previous to observation (sources: [129,130,135,138,139,140]); (**vi**) estimates of needle sharing for IDU groups in which HIV-1 prevalence was >10%, had risen fast in previous years, and the study could observe needle sharing rate at the onset of a needle exchanging program, methadone, or other intervention (known to cause dramatic drops in needle sharing) (sources: [125,128,131,132,133,136,137]); (**vii**) estimates of annual number of injections (with or without sharing) for IDUs (sources: [125,126,127,128,129,130,131,140]). For each category, the range and average between studies are shown.

Our research indicates that syphilis and yaws patients in Cameroon, in the period 1922–1931, were receiving only 1.4–2.3 injections per year (Table 1); in rural dispensaries, the average per syphilis patient could be twice that number [94]. In AEF, in the period 1930–1935, surveyed HAT patients received 3–5 injections per year, and by 1937 this had increased to 7.8 (Table 2), although newly found patients would receive around 12 injections in the first year (Section 2.4.4). In rural *hypnoseries* the average patient could receive more injections but, in the 1930s, the standard treatment was 12 injections. Reviews indicate that patients received 15–24, spread over two or more years (Section 2.4.4). Even in the unlikely scenario of these injections being all unsterile, their frequency was much smaller than either the injection frequency in modern nosocomial outbreaks, or the needle sharing frequency among IDUs (including non-sharers) suffering explosive HIV-1 spread (60–245 per year), and was comparable to the sharing frequency among IDUs with low and/or stagnant HIV-1 prevalence. A comparison of the injection intensities in Central African health campaigns, among IDU communities, and in modern HIV outbreaks can be seen in Figure 4.

### 2.5. Sexual Promiscuity and Sexually Transmitted Diseases

In the early 20th century, Central Africa suffered rampant epidemics of STDs, including genital ulcer diseases (GUD), such as syphilis and chancroid, particularly in the nascent colonial cities and socially disrupted semi-rural colonial posts. Syphilis was absent from most forested areas where chimpanzees and gorillas live, up to about 1885, when colonial control of the interior began [72,141,142,143,144,145], although it was present at the coast [72,146,147] and in savannah-forest interface regions connected to the Muslim states of the north [141]. The probable reason for this absence was the absence of commercial sex workers (CSW) with levels of sexual promiscuity comparable to those operating in the West [16,147,148,149].

In the early colonial period, administrative and trading posts were founded, with frequent steamer or railway connections to cities. Although their permanent population was no larger than that of a village, they had a high turnover of predominantly male passers-by, consisting of soldiers, traders, porters, and laborers. As happened in cities, generalized social disruption ensued in the colonial posts and enterprises located in the interior, and sex work and syphilis flourished [72,105,141,142,143,150].

Several sources mention the presence of STDs in the region since early colonial times. The Fourneau-Fondère expedition arrived to Ouesso (a French post since 1891 [151]) in 1899 with five Europeans, 36 African soldiers, and around 200 porters. Several men contracted blennorrhea and at least one contracted a chancre (of either primary syphilis or chancroid) in Ouesso, after a stay no longer than one month [152]. According to a contemporaneous reviewer, CSWs generally operated close to colonial military posts [143]. In the period 1891–1902, syphilis was already common in the Sangha posts (Ouesso, Nola, Bania), mainly driven by the movement of troops, and was still absent from interior villages [143,152]. Haoussa traders also spread STDs in the region early [153].

In the first decade of the 20th century, the French Compagnie Ngoko-Sangha and the German Gesellschaft Süd-Kamerun (GSK) intensified exploitation of local resources in the region, leading to unprecedented movements of people and social disruption. Moloundou was the administrative center of the German southeast Kamerun district, and the main GSK headquarters. The path between Yokadouma and Moloundou became frequented by thousands of soldiers and porters transporting rubber, ivory, and other products. In 1911, the number of soldiers, porters, rowers, and other African workers in the district was 1,500 (mostly young males), hired elsewhere in Cameroon, in Congo, or in other Central African colonies; the non-pygmy native population of the district was around 12,960, of whom 3,716 were adult males and 4,563 were adult females (including old adults) [54]. The sex ratio of young adults was then significantly male-biased for a rural area. Many men frequented the socially disrupted colonial posts and did not follow a rural lifestyle. Inevitably, commercial sex work became commonplace [54]. The peak intensity of a biased sex ratio and the disruption of the traditional social network probably occurred in the period 1908–1916.

We found limited direct data on the incidence of STDs and sexual promiscuity in the region of study. The high levels of social disruption and biased sex ratio suggest that STDs may have been as incident as in contemporaneous colonial troops stationed in other regions. A review of STD and GUD incidence in French colonial troops from all colonies in the period 1903–1906 indicates overall syphilis and chancroid annual incidences in the ranges 3.2%–4.1% and 5.1%–6.9%, respectively. In the more CSW-ridden Caribbean colonies these incidences were 7.3% and 13.3%, respectively [154]. Almost all syphilis detected in troops was either primary or secondary; primary syphilis is characterized by a genital chancre and secondary syphilis causes genital ulcers in a substantial fraction of patients [155].

The health reports of French Congo show the numbers of *consultants* (treated cases) for syphilis and the population partitioned between adults and children of the Ouesso (or Ngoko-Sangha) district in the period 1932–34. The reported syphilis incidence in adults in these three years was 4.8%, 5.2%, and 19.9%, respectively. The same reports show the number of *consultations* for syphilis in the Nola subdivision (which was part of the Haute Sangha district) [51]. Assuming the Nola consultations/consultants ratio is the same as in Ouesso, we can estimate the numbers of consultants in Nola; based on that we estimated syphilis incidence in Nola at 10.7% in 1933 and 15.2% in 1934. Part of these cases could be tertiary or serological manifestations of yaws, which were hard to distinguish from syphilis [51]. The very high reported incidence in 1934 may reflect an increase in treatments and serological diagnoses [51].

### 2.6. Travelling and Migration of People to/from the Stanley Pool Cities

We attempted to obtain information about migration of people from the studied area to cities, or to work in the French railway construction (1921–1932) and the Belgian railway upgrade works (1923–1931). We collected census/survey tables of colonial cities that partition inhabitants by ethnic/regional origin. These tables appear sporadically in archives, articles, and books. Some were published as supplements to our previous article [16]. For the period 1927–1934, the number of immigrants from either southeast Cameroon or the Nola area recorded in Leopoldville and Brazzaville censuses is negligible. However, these censuses likely missed floating populations and illegal immigrants, and also may have misrepresented the real origin of immigrants, so we cannot conclude that the real number was negligible. Also, we did not obtain similar censuses from before 1927. Additional research is needed to quantify these migrations in more detail.

During the colonial period the region was economically more dependent on the large southern cities of the Stanley Pool (Leopoldville-West, Leopoldville-East (Kinshasa), and Brazzaville) than on western Cameroon, because of the easy transportation highway provided by the Congo-Sangha river system. Local firms used the Belgian railway between Kinshasa and the Atlantic port of Matadi, available since 1898, to export their products. The French companies used the Belgian railway until 1932, when a second railway connecting Brazzaville to Pointe Noire was completed, diverting most of the region's exports from the Belgian railway [72,151,156,157]. In the period 1899–1914 the German connections to Kinshasa were particularly intense. The GSK was exploiting the resources of southeast Kamerun more intensively than its French counterparts in neighboring AEF, and it had large warehouses in Moloundou [54,122,151,156]. Both the GSK and the associated Kameruner Schiffahrts-Gesellschaft had steamers navigating between southeast Kamerun and Kinshasa, and Belgian archival documents refer to warehouses and other property owned in Kinshasa by these two German companies [158]. For example, on the day the First World War broke out, four German steamers were docked in Kinshasa, which illustrates the intensity of the commercial links between the city and southeast Kamerun [122]. The German administration and enterprises in Moloundou employed men described as “Bangala” [54]. This designation could mean they belonged to the Bangala tribe from the Belgian Congo (of which our previous research revealed that many men were in Kinshasa [16]), or speakers of Lingala, a trade language by then dominant in the Congo basin's colonial posts [159]; in either case they could well have frequented or inhabited the Pool cities.

After the mid-1920s the rubber business of the region declined, and coffee and cacao plantations overtook rubber in economic importance in the region (Section 2.3.3). Several important plantations were set up around Nola, Berberati, and at the Sangha banks, more than in southeast Cameroon [56]. In the 1930s, these regions probably generated more traffic to and from the southern cities than southeast Cameroon, and after 1932, when the French railway became available, this traffic was probably being increasingly directed to Brazzaville rather than to Kinshasa. In addition, the increasing importance of roads after the 1930s intensified the region's links to western Cameroon, and reduced its reliance on the Stanley Pool. Trade connections between southeast Cameroon and the Pool peaked in the period 1899–1923.

During the First World War, intense military operations occurred in the region. French forces took Nola in October 1914. Franco-Belgian forces conquered Moloundou in December and Yokadouma in January 1915. These forces totaled about 2,000 soldiers and had thousands of porters helping them. They stayed in southeast Cameroon for five months, before resuming the offensive to the west. They “encouraged” (forced?) local people to supply them with food. During these five months and beyond, through February 1916, numerous steamers supplied troops from Brazzaville and Leopoldville [122]. The numbers of soldiers and porters involved were stressful for a district with about 14,000 native people [54], and the sex ratio around the posts where the soldiers were camped became inevitably extremely male-biased. Although the war in Cameroon lasted only 18 months, it involved intense steamer movements to/from the Pool cities, intense soldier and porter movements in the interior [122,160], grave food shortages among combatants and reliance on bushmeat [160,161]. Deserters formed armed bands that roamed the region for pillage, even after the war [151]. It should be noted that both the regular troops and the bands of deserters had modern cartridge weapons, with which hunting of apes is far more effective [46] than with the muzzle-loaded guns available to most rural hunters. The war operations may have increased human exposure to SIVcpz and SIVgor, sexual promiscuity, and the probability of a zoonotic virus to arrive at the Pool cities.

During the war, German troops retreated from areas of Gabon and Congo-Brazzaville into Cameroon, and concentrated in Yaoundé and Ebolowa, the two larger urban centers that they controlled during most of the war. Invading French troops moved from the same countries into Cameroon and were stationed in the same cities when the war ended. German troops retreated to Equatorial Guinea (a neutral Spanish colony at the time), dragging tens of thousands of civilians with them. Most of these military and civilians moved back into Cameroon (passing by Douala and other cities) soon after [122,160]. More than 50,000 people were involved in these movements [122,145,160]. Thus, the war caused an unprecedented transit of people between Cameroon and neighboring regions of Congo-Brazzaville, CAR, Gabon, and Equatorial Guinea, and particularly of highly mobile people, relying on bushmeat while in the forest, and moving from forest areas to Cameroonian cities (Douala, Yaoundé, Ebolowa). At least in Douala, CSWs and GUD were abundant [50,99,105,162]. Thus, the unprecedented movements of people associated with the war may have been associated with the origins of the HIV-1 groups M and O, since both are dated from around the same time [27,28,29,30].

## 3. Discussion

As many authors suggested, HIV-1 M probably started to spread substantially in Kinshasa, whether or not initial foci started in Brazzaville or other towns or colonial posts [2,15,16,18,27]. Even though old samples from other cities have not been available up to date, not only viral genetic diversity including in old samples [27,41,42], but also phylogeographic calculations [43], and epidemiological data [38,39] favor Kinshasa as the initial epicenter of pandemic HIV-1.

SIVcpz is endemic in southeast Cameroon and the neighboring Nola area (Figure 1), where currently the SIVcpz closest related to HIV-1 M has been found (reported at sites MB, LB, and BY (Figure 1)), with a prevalence over 30% in the MB community, which is higher than the average of either central or eastern chimpanzees [2,44,45]. We show here that ape bushmeat hunting was already widespread in the region in the period 1900–1910, probably boosted by massive gun selling in the area. Local clans had a higher cultural inclination to hunt apes than elsewhere in Cameroon. Ape bushmeat hunting was probably more intense in the period 1899–1930, when the rubber business peaked, and laws protecting chimpanzees had not been enacted yet. Ape hunting was more common in southeast Cameroon and around Nola than in southwest and south-central Cameroon (Section 2.3). Considering the “patchiness” of both SIVcpz/SIVgor prevalence and ape bushmeat intensity (Section 2.1), southeast Cameroon and the neighboring Nola area might have been particularly permissive for cross-species transmission of HIV-1-like viruses. 

We also show that during the period 1899–1923, the most likely timing of the origin and initial spread of HIV-1 M, southeast Cameroon and the neighboring Nola area (Figure 1) had intense steamer traffic to/from Kinshasa (Section 2.6), surpassing neighboring AEF regions, and probably matching many regions of the Belgian Congo in this regard. Both Kinshasa and Brazzaville are located in the Stanley Pool constituting the main attraction and hub for traders, laborers, migrants, and soldiers of the giant fluvial system encompassing the navigable branches of the Congo, Oubangui, Sangha, and relevant tributaries during the colonial period. The trade relations of southeast Kamerun with Kinshasa were intense in the German period (1899–1914). At the same time, the social disruption of the region was high, with a substantially male-biased sex ratio in young adults, thousands of soldiers, porters, and workers moving between posts and to/from Kinshasa, and CSWs and STDs were present [54,70]. 

The First World War added specific risk factors that may have helped HIV-1 M to emerge. Although the war lasted only 18 months in Cameroon, thousands of soldiers, armed with modern weapons, and thousands of porters moved in the region of study, relying on bushmeat and confiscating food from local inhabitants, thus forcing them to increase their reliance on bushmeat as well. Armed bands roamed the region. Sexual promiscuity and rape are likely to have happened. Steamer traffic to/from the Stanley Pool cities also intensified [122,151]. While we want to focus here on HIV-1 M, the war hypothesis deserves further attention also for the origin and spread of HIV-1 O, which has a tMRCA roughly coinciding with that of HIV-1 M [27,28,29,30]. 

In currently observed human SIV infections, including among laboratory workers, the viral load is undetectable [19,163], suggesting lack of adaptation and transmissibility past acute infection. However, if “nascent HIV”, after the initial cross-species transmission was only transmissible among humans during acute infection, then the origin of HIV-1 M required an initial series of acute transmissions. Either the first human case (e.g., a bushmeat handler) must have carried the virus to a city during the short time frame of his/her acute infection, or there might have been some local human spread of the virus close to its geographic origin, followed by a migration to a city hub. 

In our previous work we showed that, in the early 20th century, Kinshasa had a strongly male‑biased sex ratio, the presence of CSWs, most women unmarried, a high GUD prevalence, and a lower level of male circumcision than it has today. In Kinshasa the sex ratio was more biased, sex work was more flourishing, and male circumcision was less common than in neighbouring Brazzaville [16]. We modeled the initial spread in humans of a nascent SIVcpz/HIV-1 M ancestor virus (transmissible only during acute infection) in Kinshasa [16]. Our simulations show that initial heterosexual spread of such a virus would have been much more probable under the conditions prevailing in the city in the early 20th century—particularly a high GUD prevalence [16,101]—than either in the preceding or in the subsequent periods. In our sensitivity analyses, we let the simulation parameters vary under realistic values, and we tested 51 different scenarios. GUD prevalence appeared to be a key determinant of the magnitude of the simulated epidemics and was crucial to allow for long chains of transmission [16]. In our simulations, the effects of GUD and acute infection in transmissibility combined in a multiplicative way, a method that has been used in other major published models [164,165]. Using an additive model, the effect of GUD for HIV-1 initial spread becomes less dominant [166]. However, our simulations included, for simplicity, random periods of GUD ulcerations in the affected populations (GUD transmission itself was not explicitly implemented), and thus, the real magnitude of the GUD effect may have been underestimated. We intend to upgrade our models, both the multiplicative and the additive, by implementing explicit transmission of GUD, which might generate strong correlations between GUD and HIV prevalence in the most connected parts of the sexual network and thereby intensify the effect of GUD prevalence on HIV transmission. We did not include the contribution of unsterile injections to the origin and initial spread of HIV-1 in Kinshasa in our model, but we recognize that such contribution is plausible since injection intensity was high in some of the city's health facilities in the 1920s and 1930s [18,101,167].

It has been proposed that unsterile injections given in rural Central Africa exponentially amplified HIV-1 to thousands of infections from a single SIVcpz-infected human [17,18,35], which would assign a major contribution of rural injection campaigns to the origin of HIV-1 M. In this paper, we attempted to assess the injection intensity of such campaigns around the time of the HIV-1 M origin, to have a semi-quantitative appreciation of their possible role in early HIV spread, and to have sound information on which to base future modeling efforts. We found that at present unsterile injections are not capable of sustaining an HIV-1 epidemic without extremely high rates of needle sharing, even though modern HIV-1 is probably more efficient at human-to-human transmission than the initial virus. In the general populations of modern Africa, unsterile injections are still relatively common, and they only account for an estimated 2.5% of all HIV infections [168]. HIV-1 has spread parenterally only in selected populations, such as IDUs practicing needle sharing and in hospital outbreaks. IDU epidemics are characterized by exposures of >60 unsterile injections per year (Section 2.4.7). Remarkably, even IDU populations highly infected with the Hepatitis C Virus (HCV), seem to have patchy HIV prevalence, with highly variable prevalence rates among neighboring groups [126,127,129,130,140].

Comparable levels of exposure cannot be documented in early 20th century rural Africa (Section 2.4.7). With an average injection intensity of around 12 injections per year for newly diagnosed HAT patients and less for old revisited HAT, syphilis, and yaws patients (Section 2.4.3 to Section 2.4.5), the Central African campaigns might have massively spread HCV [17,169] (as happened with anti-schistosomiasis campaigns in Egypt, which had similar injection intensity [170]), but are unlikely to have spread HIV-1 extensively [129,130,135,138,139,140]. 

During the HAT and other campaigns done in rural Central Africa in the 1920s–1940s, people formed a queue waiting for injections or vaccinations [50]. The hastiness of the procedures would leave little time for appropriate sterilization of syringes and catheters [18] and, accordingly, SIV/HIV transmissions may have occurred. However, in such procedures an infected index patient would have been able to transmit the virus to the next person in the row (the first one exposed), but hardly to the second one, because most of his/her blood remaining in the syringe would be injected in the first exposed person, and very little would remain while injecting the second exposed. Thus, the probability of infecting additional people in the queue is likely to exhibit an exponential decline, as has been observed for the probability of recovering HIV-1 from syringes after successive rinses: experiments suggest a 55%–80% decline in this probability after the first rinsing with water [171]. Per shared needle, the probability of transmission is around 0.6%–1.4% for intravenous injections and substantially less for intramuscular injections [123,125,168]. Therefore, as a thought experiment, if all people in an entire country were injected once with the same unsterilized syringe, and if there were 100 HIV-infected people before the campaign, assuming a 1% per needle share probability of transmission, the campaign would hardly do more than increase the number of infections from 100 to 101.

What is the likelihood of parenteral outbreaks of SIV/HIV in campaigns where infected index patients were injected many times (as happens in IDUs and modern hospital outbreaks)? The best candidate environments in the region of study would be the *hypnoseries* (such as in Nola and Ouesso) where the more severe HAT patients might have been interned together and could have received more than the standard number of 12 injections. If an SIV-infected index case entered such a ward, the situation might have mimicked modern nosocomial outbreaks with the virus being able to spread locally, adapt to humans, and possibly initiate an epidemic in a city. This scenario could have produced a local outbreak, but not a massive outbreak to thousands of people [17,18,35].

A rural hospital/*hypnoserie* outbreak would be plausible if, in such a rural facility, the frequency of boiling or rinsing of syringes was low, and the injection intensity was much higher than the average for HAT (Table 2). The probability of such conditions would have been higher after the early 1930s for several reasons: (1) the first systematic HAT, syphilis, and yaws campaigns started in 1932 in southeast Cameroon, and in 1930 in the Nola sector (Table 3; Section 2.4.5); (2) before that, intravenous drugs such as *tryparsamide* were less commonly used (subcutaneous/intramuscular *atoxyl *predominated). Thus, the parenteral risks in the region of study strongly intensified after 1930–32 (Section 2.4.5). If HIV-1 had spread intensively in rural areas by unsterile injections in the 1930s, it would likely have continued to do so in the 1940s and 1950s, when injection intensity was higher (Figure 2). One would expect the postulated thousands of people so infected [17,18,35] to have maintained this rural HIV-1 focus in the region for decades. However, the several serological surveys that were made in the area in the period 1975–1992 found zero prevalence until 1987, when HIV-1 arrived there with the pandemic wave (reviewed in [38]).

Whereas recent datings of the origin of HIV-1 M (corroborated by the use of two “fossil” virus samples from 1959 and 1960) calculated the tMRCA of HIV-1 M in the range 1900–1915, with 95% confidence interval 1873–1924 [27,30], it cannot be excluded that these dates may still change, for example if heterotachy (differing evolutionary rate between lineages [30,172]) results in overestimating the age of HIV-1 M [30]. In addition, previous datings [25,26] included the 1930s in their 95% confidence interval. Further refinement of molecular dating techniques, and the discovery of new HIV-1 M “fossil” viruses may increase the accuracy of tMRCA datings and, when this happens, all arguments will have to be revised in the light of such new findings.

The hypothesis of HIV having adapted through a parenteral serial passage event [4,32,33,34] cannot be ruled out, given that many injections and smallpox vaccinations happened in the region of study, even before 1930 (Section 2.4.2 to Section 2.4.4). However, this is not a unique feature of parenteral serial transmission, and could perhaps more easily have happened by sexual serial transmission (or by a combination of sexual and parenteral transmission). Since the late 1890s there were CSWs, gonococcal urethritis, syphilis, and possibly other GUDs in the colonial posts of the region [51,54,152] (Section 2.5). Sex acts involving GUD have higher HIV transmission probability than unsafe intravenous injections: for a female with a genital ulcer, the per-act female-to-male transmission risk was estimated to be 4% if the male is circumcised and 43% if he is uncircumcised [36,37], while the male-to-female risk was estimated to be 7% [37]; these values are substantially higher than the estimates of per-needle share transmission risks, which fall in the range 0.6–1.4% [123,125].

The most recent tMRCA calculations fit well with the time of most intense selling of firearms in the region (1900–1907), maximal rubber exploitation in the region (1899–1923), intense trade relations with Kinshasa (1899–1932), First World War (1914–1916), the period when chimpanzee hunting was not outlawed (pre-1930), and the period of intense GUD epidemics in Kinshasa [16]. If the tMRCA calculations are correct, the above factors may have been more important for HIV-1 M origin than unsterile injections, which had a relatively low frequency in the region before 1930–1932.

The coincidence of the above referred factors in a short period of time, together with the high SIVcpz prevalence in the wild, and the local peoples’ penchant for ape hunting (Section 2.3), may indicate that the origin of HIV-1 M was opportunistic, more determined by the conjunction of favorable factors and opportunities, than by any (unknown) superiority of the local SIVcpz viruses over others in their *a priori *genetic ability to infect humans. Further biological studies are however needed to clarify this. We are not suggesting here that the studied region was unique, just that the superimposition of the three geographically patchy variables (ape SIV prevalence, ape bushmeat intensity, and intensity of contacts with the Stanley Pool cities) probably implied that, in the whole range of *P.t.troglodytes*, *P.t.schweinfurthii*, and *G.g.gorilla*, few hot spots with comparably high risk of HIV emergence existed, and that the region under study was one of such hot spots. Why epidemic HIV-1 arrived to Kinshasa only from that hot spot and not from others might have been a matter of chance. Our suggestion that local high SIVcpz prevalence (as observed by recent studies) was relevant to explain the location of HIV-1 M origin depends on the assumption that regional differences in SIVcpz prevalence did not change dramatically in the *P.t.troglodytes* range in the last 100 years. Although this assumption is not proven, it is parsimonious, and in the absence of contrary evidence, we consider it reasonable.

We found no clear evidence of parenteral risk factors having played a decisive role in HIV adaptation and emergence, and we found that intense co-factors potentially favoring HIV heterosexual transmission were present in both the region of study and in the Stanley Pool cities at the likely time of origin. Therefore we propose an Enhanced Heterosexual Transmission Hypothesis for the origin of the several HIV groups: each of these viruses emerged and adapted to humans through heterosexual transmission enhanced by a spatial and temporal coincidence of a high intensity of factors that favor cross-species transmission (SIV prevalence, ape bushmeat intensity), and of the same co-factors of heterosexual transmission that are today observed to contribute the most to the current African epidemic, without the need to invoke additional factors. Since we are proposing a major role for co-factors of heterosexual transmission, which is the overwhelmingly predominant mode of transmission in Africa, our hypothesis follows the principle of parsimony. Our data shows that the coincidence of factors existed for HIV-1 M and we hypothesize that it permitted the virus to emerge by heterosexual transmission. Our hypothesis predicts that similar coincidence is likely to have been a key element in the origin of the other epidemic HIV-1 and HIV-2 groups, as well.

The Enhanced Heterosexual Transmission Hypothesis rests not only on patterns of sexual behavior, but also on other factors that influence HIV transmission by the heterosexual route. The Four Cities Study [173] found that differences in HIV prevalence between four African cities (including Yaoundé) were best explained by patterns of male circumcision (and not by patterns of sexual promiscuity). The Enhanced Heterosexual Transmission Hypothesis includes that the lower historical prevalence of circumcision in the early 20th century (compared with the current situation) in Central Africa [16] probably contributed to the emergence of pandemic HIV-1. Furthermore, sexual promiscuity might have played a stronger indirect role than today by fuelling rampant GUD epidemics, which very strongly facilitate the transmission of HIV but are, today, in contrast to the early 20th century, controlled by treatments in spite of high promiscuity.

Our research shows how varied the risk factors present in the region of origin of HIV-1 M were, and how far we are from knowing the complete picture. Either sexual or parenteral serial transmission could have partially adapted the virus in the studied region. It could have generated a small local epidemic before moving to Kinshasa, or it could have been brought there by a migrant bushmeat handler directly. Further sampling of SIVcpz viruses from many sites within the Boumba-Ngoko-Sangha enclosure and more migration information during the last century from its host, the chimpanzee, could help to establish better where the virus crossed to humans, which in turn could help to test the hypotheses above exposed. Refinement of MRCA dating techniques and new “fossil” viruses could establish more clearly whether increasing parenteral risks after 1932 were accompanied by a radiation and expansion of the virus. Further archival research might help to quantify trade and migration ties to Kinshasa, Brazzaville, and other cities, the intensity of GUDs in the region, and to investigate the possible role of World War I. New simulations could test competing hypotheses. What exactly happened at the origin of HIV-1 M and HIV-1 O is still mostly unknown.

## 4. Sources Used

In addition to the listed sources about HIV, SIV, and other retroviruses, we consulted major reviews about the relevant epidemics of tropical diseases and STDs discussed here (sources: [17,18,34,39,40,72,102,115,116,117,118,119,120,121]). We obtained quantitative data about diseases and their treatments for the whole of Cameroon, AEF, and for the districts of these countries situated in the region of study from:

(1) Official colonial reports about health, or containing sections about health, and covering also demographic issues. (A) For AEF and Cameroon, period 1920–1960, collected in the Archives Nationales d’Outre-Mer (ANOM), Aix-en-Provence (France), and in the Institut de Médecine Tropicale du Service de Santé des Armées (IMTSSA), Marseille (France). (B) For Cameroon, we used also the reports from the French administration to the League of Nations [50]. (C) For Kinshasa and the Belgian Congo we consulted the Afrika Archief, Federale Overheidsdients—Buitenlandse Zaken, Buitenlandse Handel en Ontwikkelingssamenwerking (FO-BZBHO), Brussels (Belgium).

(2) We complemented this information with articles in the main colonial and tropical medicine journals (Ann Méd Pharm Coloniales, Ann Hyg Méd Coloniales, Bull Soc Pathol Exotique), and additional articles and books on the subject. Particularly important was a series of annual articles in Ann Méd Pharm Coloniales, covering health data in French colonies and territories under administration in the period 1927–1936 [87,88,89,90,91,92,93,94,95,96,97].

We collected primary ethnographic, primatological, and other works to obtain information about chimpanzee and gorilla hunting in the region under study, including legislation [46,47,48,54,55,56,57,58,59,60,61,62,63,64,65,66,67,68,71,73,74,75,76,77,78,79]. For this region, these sources offer a more detailed picture than the larger set of sources we used in our previous literature survey (applying to Central Africa as a whole), listed in the main and supplementary reference lists published with our previous article [16]. We complemented these with additional sources about the modern bushmeat crisis and ape population decline.

We consulted historical and anthropological sources about social, economic, demographic, and military issues, most of which directly cover the region under study [54,55,56,67,68,69,70,122,141,145,151,152,153,156,157,158,160,162].

We consulted modern sources about IDUs, focusing on their needle sharing behavior, and prevalence of HIV-1 and HCV [125,126,127,128,129,130,131,132,133,134,135,136,137,138,139,140], and reviews of modern HIV-1 nosocomial outbreaks [123,124,168].

## 5. Conclusions

In this article, we reviewed the factors that may have helped HIV-1 M to emerge from its birthplace in the African rainforest. The cross-species transmission from chimpanzees has been suggested to have taken place in southeast Cameroon or the neighboring AEF areas. Indeed, SIVcpz is far more prevalent in this area than in most other areas of the *P.t.troglodytes* and *P.t.schweinfurthii* range, while SIVgor has not been found there. The conjunction of favorable conditions in a short period of time—ape bushmeat hunting intensification, social disruption, nascent cities with commercial sex workers and STDs (including genital ulcer diseases)—seem to have coincided with tMRCA estimates for HIV-1 M, suggesting that the origin of the epidemic was opportunistic, probably more associated with these conditions than with a potential predisposition of local SIVcpz over other SIVcpz to infect humans.

While the parenteral hypothesis of HIV origins still deserves further verification through modeling, we already see two major difficulties. First, the injection treatments in the region intensified sharply only in 1930–1932, while the upper confidence intervals of most recently published tMRCA calculations fall before that. Second, local syphilis, yaws, and HAT patients received far fewer injections per year than current IDUs and patients in hospital outbreaks for which good records were kept. Therefore, the strongest version of the parenteral theories, that proposing massive parenteral spread of HIV-1 to thousands of people in rural areas, seems unlikely. A more limited local parenteral outbreak of HIV-1 could have occurred in *hypnoseries* or other health facilities where injection intensity was higher than average, especially after 1932. The hypothesis of HIV-1 adaptation by serial passage involving a short chain of transmissions is plausible and could have happened at any time during colonial domination of the region. This mechanism could have happened by either parenteral or sexual transmission. 

We therefore propose an Enhanced Heterosexual Transmission Hypothesis for the origin of HIV-1 M, by which the emergence of the virus was caused by a coincidence of high intensity of favorable factors for cross-species transmission (SIV prevalence and ape bushmeat hunting), and of factors that are today associated with HIV heterosexual spread in Africa (travel/migration, social disruption, sex work, STDs, GUDs, and possibly lack of male circumcision). While there is no necessity for parenteral transmission in this hypothesis, it does not exclude a role for it in the initial adaptation of SIVcpz to humans.
